# A pilot study evaluating concordance between blood-based and patient-matched tumor molecular testing within pancreatic cancer patients participating in the Know Your Tumor (KYT) initiative

**DOI:** 10.18632/oncotarget.13225

**Published:** 2016-11-08

**Authors:** Michael J. Pishvaian, R. Joseph Bender, Lynn M. Matrisian, Lola Rahib, Andrew Hendifar, William A. Hoos, Sam Mikhail, Vincent Chung, Vincent Picozzi, Craig Heartwell, Kimberly Mason, Katelyn Varieur, Metasebia Aberra, Subha Madhavan, Emanuel Petricoin, Jonathan R. Brody

**Affiliations:** ^1^ Perthera, Inc, McLean, VA, USA; ^2^ Lombardi Comprehensive Cancer Center, Georgetown University Medical Center, Washington, DC, USA; ^3^ The Pancreatic Cancer Action Network, Manhattan Beach, CA, USA; ^4^ Cedars-Sinai Medical Center, Los Angeles, CA, USA; ^5^ Ohio State University, Columbus, OH, USA; ^6^ City of Hope Cancer Center, Duarte, CA, USA; ^7^ Virginia Mason Medical Center, Seattle, WA, USA; ^8^ Jefferson Pancreatic, and Related Cancer Center, Kimmel Cancer Center, Thomas Jefferson University, Philadelphia, PA, USA

**Keywords:** cfDNA, pancreatic cancer, blood-based NGS

## Abstract

Recent improvements in next-generation sequencing (NGS) technology have enabled detection of biomarkers in cell-free DNA in blood and may ultimately replace invasive tissue biopsies. However, a better understanding of the performance of blood-based NGS assays is needed prior to routine clinical use. As part of an IRB-approved molecular profiling registry trial of pancreatic ductal adenocarcinoma (PDA) patients, we facilitated blood-based NGS testing of 34 patients from multiple community-based and high-volume academic oncology practices. 23 of these patients also underwent traditional tumor tissue-based NGS testing. cfDNA was not detected in 9/34 (26%) patients. Overall concordance between blood and tumor tissue NGS assays was low, with only 25% sensitivity of blood-based NGS for tumor tissue NGS. Mutations in KRAS, the major PDA oncogene, were only detected in 10/34 (29%) blood samples, compared to 20/23 (87%) tumor tissue biopsies. The presence of mutations in circulating DNA was associated with reduced overall survival (54% in mutation-positive versus 90% in mutation-negative). Our results suggest that in the setting of previously treated, advanced PDA, liquid biopsies are not yet an adequate substitute for tissue biopsies. Further refinement in defining the optimal patient population and timing of blood sampling may improve the value of a blood-based test.

## INTRODUCTION

Pancreatic ductal adenocarcinoma (PDA) is an aggressive cancer that is projected to become the second leading cause of cancer-related death by 2025 [1]. This is, in part, due to poor early detection strategies: most cases are detected at an advanced stage [2] despite the long amount of time required for metastatic disease to develop [3]. However, even resectable pancreatic cancers usually recur [2]. The current standard of care therapies for metastatic disease are comprised of cytotoxic chemotherapies, but despite recent improvements, the median overall survival remains less than one year [4, 5]. In theory, personalized therapy for PDA promises a more rational approach than “standard of care” treatment, by identifying and targeting “actionable” or “driver” mutations.

With the advent of new commercially available CLIA/CAP accredited lab testing for next-generation sequencing (NGS) panels, detection of actionable mutations from tissue biopsies no longer requires that the patient be seen at a specialized high-volume tertiary care academic medical center. Still, potential obstacles in detecting mutations from patient samples include the tumor not being accessible via biopsy and/or not enough tumor cells being extracted for DNA analysis. A potential solution for these problems is the development of “liquid biopsy” techniques that use the same NGS technologies for molecular profiling. Detection of circulating tumor DNA (ctDNA) in cell-free DNA (cfDNA), circulating tumor cells (ctcDNA), and tumor exosome-containing genomic material has created the possibility of a non-invasive method for diagnosing and monitoring cancer [6].

We previously launched an initiative (Know Your Tumor, a collaboration between Perthera and the Pancreatic Cancer Action Network), which includes multi-omic molecular profiling of PDA patients’ tumors and matches patients with appropriate clinical trials and therapies based on actionable molecular anomalies, treatment history and geographical locations. However, to effectively implement this precision medicine strategy, biopsy samples with relatively high levels of tumor cells are needed, forcing us to exclude PDA patients with locally advanced disease, or those with small volume, non-biopsiable disease. As a promising solution, we evaluated two CLIA/CAP accredited blood-based NGS assays as a potential substitute for gold standard tissue biopsy procedures. Here we describe our experience with a pilot study of 34 PDA patients that represent a “real world” setting of consecutive patients with metastatic, disseminated disease in community and academic settings from locations across the United States.

## RESULTS

### Feasibility of performing blood-based NGS assays from PDA patients regardless of the clinical setting

To evaluate the feasibility of incorporating circulating DNA-based NGS assays into a precision medicine strategy for pancreatic cancer, blood-based NGS assays were performed on 34 patients. Blood draws were sent to commercial laboratories for NGS analysis by the Guardant Health Guardant360 test (*n* = 26) or the Cynvenio ClearID test (*n* = 8). Hereafter, these assays will be referred to as the cfDNA-based NGS assay and the ctcDNA-based NGS assay, respectively. The majority of patients had extensive disease burden that had metastasized to the liver, lung, or peritoneal cavity. However, several patients had either localized disease or no detectable disease due to distal pancreatectomy or Whipple procedure (Table 1 and Table S1). In 57% of cases (13/23), blood samples were collected within six weeks of the tumor biopsy, and, importantly in 74% of cases (17/23), blood samples were collected while the patient's clinical condition (extent of disease, and response to therapy) had not changed since the tumor biopsies (Table S1).

**Table 1:** Patient characteristics

	cfDNA-based NGS (*N* = 26)	ctcDNA-based NGS (*N* = 8)
**Gender**		
Male	13	5
Female	13	3
**Age, years**		
Median	66.5	63
Range	48 – 83	30 – 74
**Disease Burden**		
Extensive	17	7
Localized/Minimal	6	0
None	3	1
**Status at Blood Draw**		
Progressing	10	5
Stable	9	3
Responding	7	0
**Tumor Biopsy Site**		
Liver	9	5
Pancreas	2	1
Lung	2	1
Peritoneum	2	0
Duodenum	1	0
**Treatment Setting**		
Hospital	19	8
Community Practice	7	0

Tumor biopsy sites are only listed for patients with tumor tissue NGS data. Patients with disease burden listed as “None” were those that had undergone pancreatectomy or Whipple procedures and had no evidence of recurrence.

### Concordance between blood-based and tumor tissue biopsies

**Figure 1:**
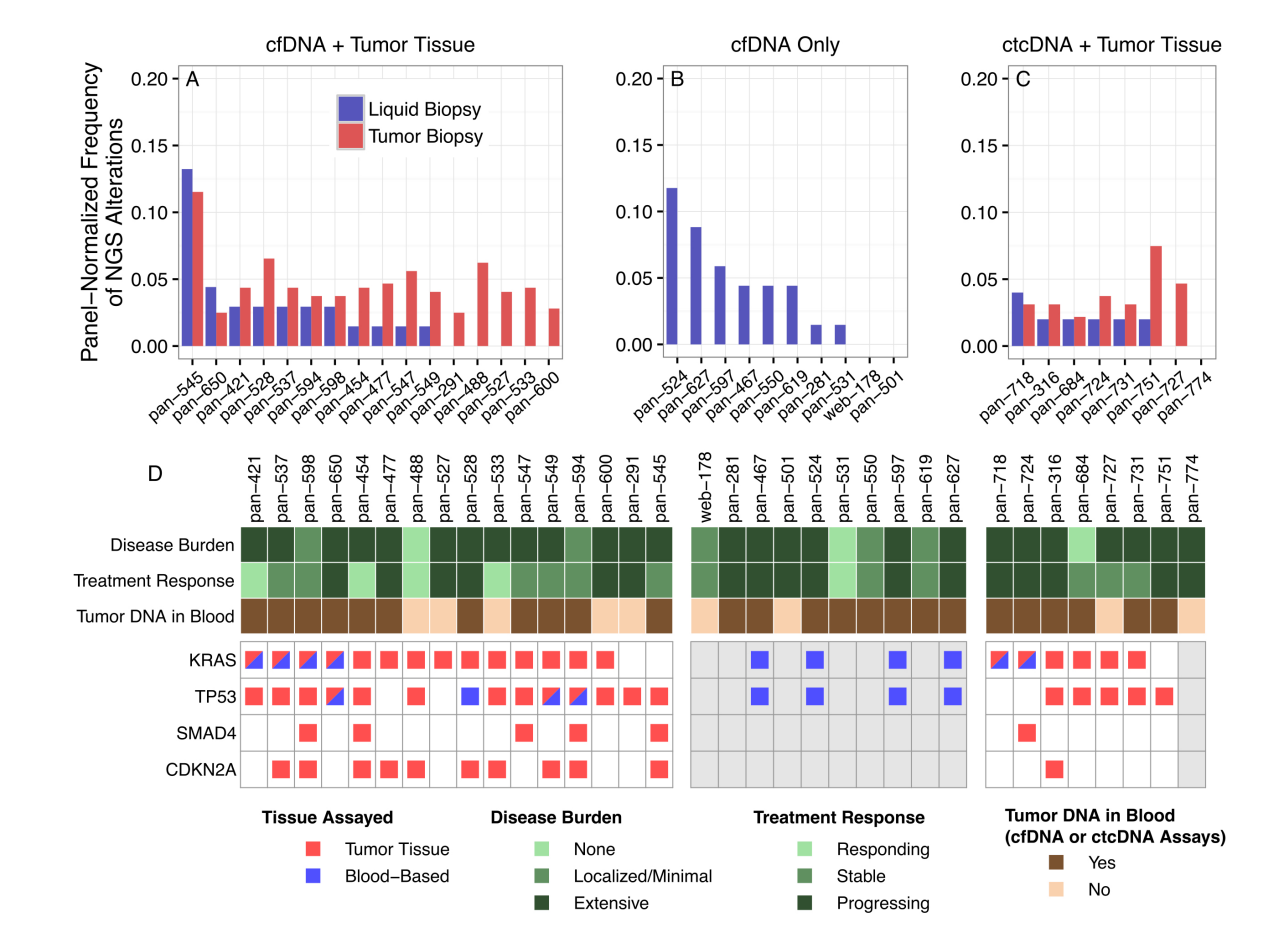
Panel-normalized number of alterations detected in liquid and tumor tissue biopsies **A.** On average, more variants were detected in the tumor tissue biopsy (red) than the cfDNA-based assay (blue) in patients with data from both assays available. Panel normalization was performed by dividing the number of mutations detected in each patient by the number of genes on the panel (*N* = 321 for tumor tissue, *N* = 68 for cfDNA, and *N* = 50 for ctcDNA). **B.** The number of variants detected in the patients for whom only the cfDNA-based assay was performed was similar to that of the cfDNA-based assays in panel A. **C.** More variants were detected in the tumor tissue biopsy (red) than the ctcDNA-based assay (blue). No tumor tissue biopsy was available for the last patient listed, pan-774. **D.** The pancreatic cancer driver genes *KRAS*, *TP53*, *SMAD4*, and *CDKN2A* were detected less frequently in cfDNA-based biopsies. Patients are subdivided according to which biopsies were performed: patients for whom both cfDNA and tumor tissue biopsies were obtained are in the left block, while patients for whom only cfDNA biopsies were obtained are in the middle block, and patients with both ctcDNA and tumor tissue biopsies are in the right block. Patients for whom tumor quantity was insufficient for tissue-based NGS are shaded in gray. Disease burden and treatment response were determined based on the most recent CT scans prior to drawing of blood samples.

In the 19 patients with detectable tumor mutations in cfDNA, mutations were found in a median of 2 genes per patient (Figure 1 and Table S2). This number was lower than tumor tissue biopsies, in which a median of 13 genes per patient had mutations. A probable reason for the lower number of mutations in the cfDNA-based assay is that the panel had 68 genes while the panel used for tumor tissue NGS had 321 genes. After normalizing by the number of genes on each panel, the median frequency of altered genes per panel was similar (2.9% for the cfDNA-based NGS assay and 4.0% for the tissue-based NGS assay). However, in general there was no correlation between the number of mutations found in cfDNA and in tumor tissue (Figure 1A). We therefore focused on analyzing concordance in overlapping genes.

**Table 2:** Comparison of concordance in published liquid biopsy studies

	Zill et al. 2015 [11] (*N* = 26)	Lanman et al. 2015 [10] (*N* = 165)	Bettegowda et al. 2014 [23] (*N* = 206)
**Patient Characteristics**			
Cancer Type	18 PDA 8 Biliary	57 Colorectal 22 Other GI 86 Other	206 Colorectal
Stage	3 Stage III 23 Stage IV	40 Stage III 120 Stage IV 5 Unknown	206 Stage IV
Tumor Biopsy Site	11 Primary 15 Metastasis	Unspecified	Unspecified
**Info**			
Assay	cfDNA	cfDNA	cfDNA
Commercial Lab	Guardant Health	Guardant Health	PGDx
**Liquid-Tumor Comparison**			
Overall Sensitivity	92.3%	85%	Unspecified
*KRAS* Sensitivity	100%	88%	87.2%

### Detection of KRAS mutations by blood-based NGS

We analyzed concordance between blood-based and tumor tissue biopsies in the 23 patients that had both blood-based and tumor tissue NGS analyses. We first examined four of the most frequently altered genes in pancreatic cancer, *KRAS*, *TP53*, *CDKN2A*, and *SMAD4* (Figure 1D). In the patients with both blood-based and tumor tissue NGS, nine (39%) were concordant for *KRAS* status (6 mutants and 3 wild-type). The blood-based NGS assays did not detect 14 *KRAS* variants (61%) that were present in the tumor tissue. We noted that in patients for whom *KRAS* mutations were detected in both tumor tissue and blood, all tumor samples were biopsied from liver metastases (Table S4). The low detection rate of *KRAS* mutations in circulating DNA is problematic since this gene is mutated in over 90% of PDA tumors in most reports [7, 8].

To determine whether technical limitations played a part in the low rate of detection of *KRAS* mutations plasma cfDNA, we examined the sequencing quality metrics where possible. The median sequencing coverage for *KRAS* mutations in tumor tissue sequenced using the FoundationOne panel was 845x, with only one sample below 500x, in line with the analytical validation study published by Foundation Medicine [9]. Quality control metrics were not obtainable for the Guardant360 assay, but validation of the assay has demonstrated a depth of coverage of 8, 000X and a limit of detection of 0.25% [10]. For the ClearID assay, the cell-free DNA yield from the plasma samples (*n* = 8) ranged from 1.2 to 10 ng. Of the 4 patients with tumor tissue *KRAS* mutations but no cfDNA *KRAS* mutations, one of them had a low cell-free DNA yield (1.7 ng), while the other three had high yields (>8 ng) that were similar to the two patients with *KRAS* mutations detected in both plasma and tumor tissue. This indicates that both technological (low cfDNA yields) and biological (actual lack of *KRAS* mutations in the plasma) limitations likely play a role.

### Identification of well-established PDA drivers in cfDNA analysis

In the 23 patients with both blood-based and tumor tissue NGS, six (26.1%) were concordant for *TP53* status (3 mutants and 4 wild-type). The blood-based NGS assays did not detect 15 *TP53* variants (65.2%) that were present in the tumor tissue. The three concordant *TP53* variants were G325*, V272L, and R273C (Figure S1). Three patients had differing *TP53* mutations in cfDNA-based and tumor tissue NGS: pan-545 (G389G in cfDNA, S261fs*2 in tumor), pan-594 (H193L and V272L in cfDNA, only V272L in tumor), and pan-598 (P278S in cfDNA, P153fs*28 in tumor).

No mutations in *CDKN2A* or *SMAD4* were detected in the blood-based assays (Figure 1D). Mutations in these genes were detected in tumor tissue in 11/23 (47.8%) and 6/23 (26.1%) patients, respectively. A possible reason for the absence of these variants in the blood-based NGS analysis is that the assays are not validated for gene deletions, indels, or splice site mutations. Many of the *CDKN2A* (8/11, 73%) and *SMAD4* (4/6, 67%) variants detected by tissue-based NGS were deletions, indels, or splice site mutations and thus would not be expected to be detected by the blood-based NGS assays.

### Sensitivity and precision of cfDNA-based NGS for tumor biopsy mutations

To systematically assess the performance of blood-based NGS assays across all genes, we determined the sensitivity and specificity of the cfDNA-based assay using the tumor tissue NGS data as the reference. For this analysis, we only considered the patients for whom both cfDNA and tumor tissue NGS data were available. Also, to be consistent with methodologies described previously [10, 11], we only considered the patients in which tumor DNA was actually detected in cfDNA, reducing the number of patients under consideration to 11. We removed a number of alterations from the tumor tissue NGS data prior to assessing sensitivity and precision: we removed any gene not included on the cfDNA assay panel; we removed deletions, indels, and rearrangements (the cfDNA panel only detects point mutations and amplifications); we removed tumor tissue amplifications for genes whose copy number could not be determined by the cfDNA assay (amplifications can only be assessed for a subset of genes on the cfDNA panel); and we removed tumor tissue mutations in exons not covered by the cfDNA panel. After these filters were applied, the remaining dataset consisted of 55 alterations across 25 genes.

**Figure 2:**
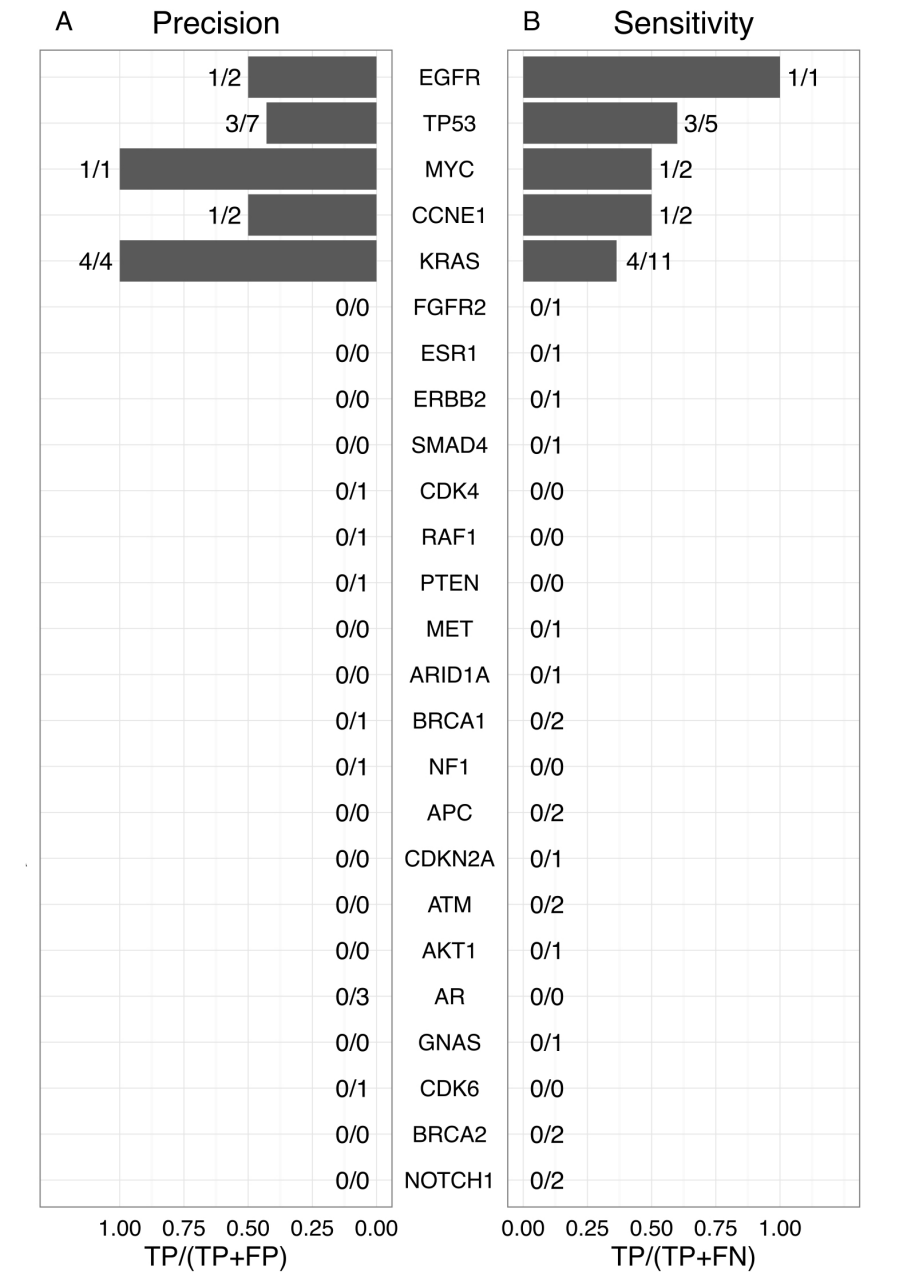
Precision and sensitivity of cfDNA-based NGS assay in detection of tumor tissue variants The precision (**A**) andsensitivity (**B**) were calculated for each gene listed in the middle using the formula at the bottom of the respective panel. TP indicates the number of true positives, or concordant variants; FP indicates false positives, or the number of variants in cfDNA but not present in tumor tissue; FN indicates false negatives, or the number of variants in tumor tissue but not in cfDNA.

The precision and sensitivity of the cfDNA-based NGS assay could only be evaluated for the five genes for which concordant variants occurred; the remaining 20 genes were only observed in one of the two assays. Precision, defined as the proportion of cfDNA-based mutations that were detected in both assays, was 100% for *KRAS* and *MYC* (Figure 2A), although there was only one *MYC* alteration. Sensitivity, defined as the proportion of tumor tissue-based mutations that were detected by both assays, was 100% for *EGFR* (Figure 2B), although there was only one *EGFR* alteration. The remaining genes had low values of sensitivity and precision. The high precision/low sensitivity for *KRAS* indicated that a *KRAS* variant detected by the cfDNA-based NGS assay is likely present in the tumor tissue, although the absence of a *KRAS* variant in cfDNA does not necessarily imply a lack of *KRAS* mutations in tumor tissue. The overall precision and sensitivity across all 25 overlapping genes were 40% and 25%, respectively.

### Therapeutic implications for assays tested

**Figure 3:**
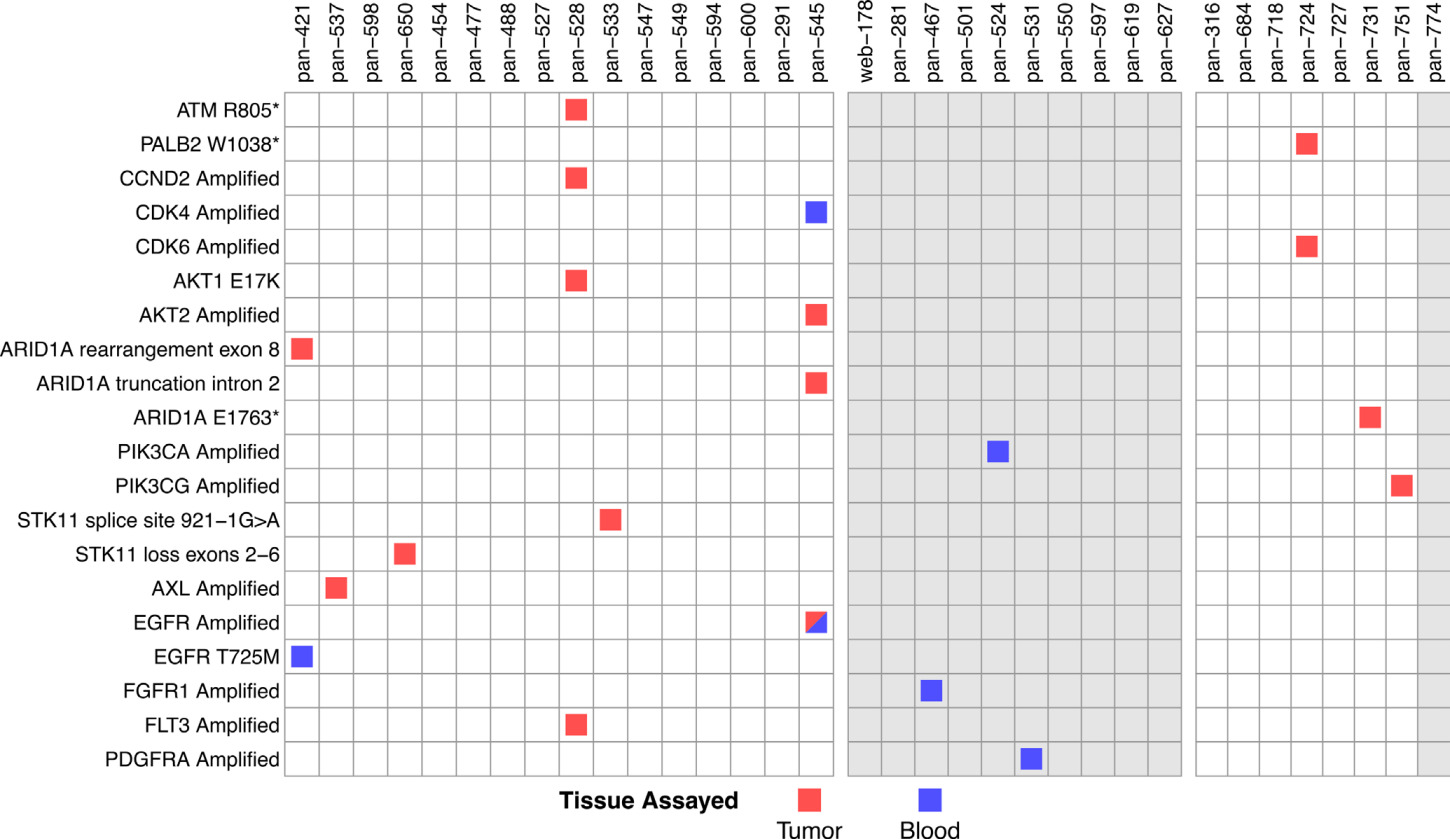
Detection of actionable mutations Variants with therapeutic implications were detected in both tumor tissue and cfDNA. Patients are subdivided into those with tumor tissue and cfDNA data (left block), cfDNA only (middle block), and tumor tissue and ctcDNA (right block). Variants found in tumor tissue NGS analysis are indicated by a red square, variants found in cfDNA-based NGS analysis are indicated by a blue square, and the sole concordant variant is indicated by a half-red/half-blue square. Patients for whom tumor quantity was insufficient for tissue-based NGS are shaded in gray.

We examined mutations that confer drug sensitivity to explore differences in the therapeutic implications derived from cfDNA-based and tumor tissue-based NGS assays. These actionable mutations included *ATM* and *PALB2*, which may indicate sensitivity to PARP inhibitors [12] or platinum agents [13]; *CCND2*, *CDK4*, and *CDK6*, which may indicate sensitivity to CDK inhibitors [14, 15]; *AKT1*, *AKT2*, *ARID1A*, *PIK3CA*, *PIK3CG*, and *STK11*, which may indicate sensitivity to PI3K/mTOR inhibitors [16]; and the receptor tyrosine kinases *AXL*, *EGFR*, *FGFR1*, *FLT3*, and *PDGFRA*, which may indicate sensitivity to various tyrosine kinase inhibitors [17–21]. Consistent with the low overall concordance we observed between cfDNA and tumor tissue NGS data, only one actionable mutation was detected in the same patient by both assays, an *EGFR* amplification in patient pan-545 (Figure 3).

In ten patients with cfDNA analysis and no tumor tissue biopsies, three actionable mutations were found (Figure 3, middle panel). In eight patients with ctcDNA analysis, tumor tissue-based NGS revealed four actionable markers, none of which were detected by the ctcDNA assay (Figure 3, right panel). These discrepancies between tumor tissue-based and blood-based NGS assays indicate that further technical improvements are needed before blood-based NGS assays can be successfully applied in therapeutic decision-making.

### Impact of clinical variables on the detection of tumor DNA in plasma

**Figure 4:**
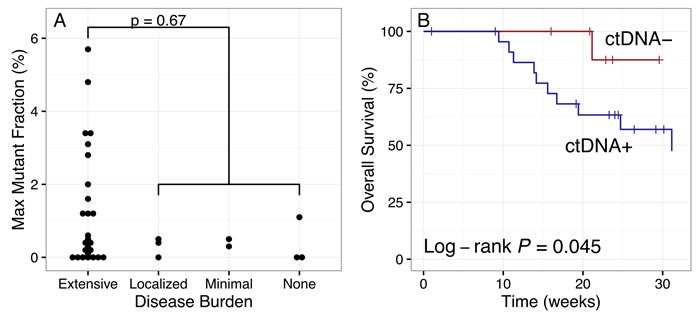
Detection of tumor DNA in circulating DNA and prognostic significance in pancreatic cancer patients **A.** The maximum mutant allele fraction in cfDNA or ctcDNA trended higher in patients with extensive disease, although this was not statistically significant. **B.** Overall survival trended lower in patients with detectable tumor DNA in cfDNA (ctDNA+, *n* = 24) than in those with no detectable tumor DNA (ctDNA-, *n* = 10), with a total of 11 and 1 deaths, respectively.

Mutations in circulating DNA were only detected in blood-based NGS analysis of 25 (74%) of the patients: 19 out of 26 (73%) by the cfDNA-based assay and 6 out of 8 (75%) by the ctcDNA-based assay. Since one of our goals is to detect actionable mutations that can influence treatment decisions, it is critical to know when a blood-based assay is most likely to detect specific tumor DNA mutations. Therefore we explored the clinical factors related to the presence or absence of detectable mutations in the blood. We used the maximum variant allele fraction in the blood-based assays as a surrogate for level of tumor DNA in the blood [10]. The patients with the six highest frequencies of somatic alterations in circulating DNA had extensive disease that was present in both the pancreas and distal metastases (Figure 4A). However, ten patients with extensive disease had no detectable tumor DNA in the blood. Several other clinical variables, including current treatment response status, time since most recent treatment, time since diagnosis, and type of most recent therapy, were not correlated with the presence of mutations in circulating DNA (Figure S3). There was a weak correlation (Pearson's *R* = 0.41) between the level of CA19-9 marker and the maximum variant allele fraction (Figure S3C). While a larger sample size is needed to draw definitive conclusions, these results indicate that clinical covariates may lack strong associations with the presence or absence of mutations in cfDNA and therefore may not be sufficient indicators of whether a blood-based NGS assay will reliably detect tumor mutations.

### Correlation of cfDNA mutations with prognosis

Although the disease burden did not appear to be strongly predictive of the presence of mutations in circulating DNA, the presence of mutations did have prognostic significance. Outcome data was available for all patients with a median follow-up (time since blood sampling) of 28 weeks. Overall survival was lower in the subset of patients in which mutations were detected in circulating DNA (*n* = 24), with 11 deaths occurring in this subgroup compared to one in the subgroup with no detectable tumor DNA (*n* = 10) (Figure 4B, log-rank *P* = 0.045). The prognostic significance of the presence of mutations in the circulation highlights a possible role for blood-based NGS in clinical care of pancreatic cancer patients, in keeping with the recent role for CTC analysis in monitoring disease burden [22].

## DISCUSSION

Despite the promise of noninvasive liquid biopsies, our data suggest that circulating DNA-based NGS assays do not yet appear ready to replace tumor tissue biopsies in detecting actionable mutations for use in pancreatic cancer precision oncology strategies. We analyzed a pilot study set of consecutively enrolled patients with metastatic PDA who were enrolled in a “real-world“ community setting, which represents the exact type of target patient population that would be most impacted by these evolving molecular practices given the high percentage of metastatic cancer patients treated at the community level. The low concordance between cfDNA-based and tumor tissue NGS assays in our data would yield lower numbers of actionable mutations if only the cfDNA-based assay data were available. Given the high frequency of *KRAS* mutations in PDA of over 90% in most reports [7, 8], we can use this specific genomic alteration as a gold standard to gauge technical utility, which provides a unique window into cfDNA-tumor tissue concordances. Our results showed poor sensitivity of cfDNA blood-based testing to identify *KRAS* mutations. However, we did find evidence for potential clinical utility based on the prognostic significance that we observed (Figure 5B).

Several performance characteristics of blood-based NGS analysis were similar in our study and other published studies that used the same commercial cfDNA-based NGS assay. Mutations in blood were detected in 73% of the cfDNA-based NGS assays here, while in other studies this frequency was 86% [10] and 96% [11]. We found a median of 2 mutated genes per patient in the cfDNA-based NGS assay, while other studies found means of 3.3 [10] and 2.8 [11] altered genes per patient.

A major difference between our data and other studies is the very low concordance of genomic alterations found between liquid and tumor tissue biopsies (Table 2). One study of 165 patients demonstrated 85% sensitivity and 80.7% precision in detecting tumor tissue variants using cfDNA-based NGS [10]. Another study involving 17 pancreatic and biliary cancer patients reported an overall sensitivity of 90.3% and an overall precision of 87.5% [11]. A highly specific digital PCR method has been used to sequence the plasma cfDNA of pancreatic cancer patients with known *KRAS* mutations, finding *KRAS* mutations in the plasma of 30/34 (88%) patients with metastatic disease and 59/121 (49%) patients with localized disease (precision could not be evaluated because all patients had *KRAS* mutations) [23]. Our sensitivity and precision were much lower, indicating that many mutations would be missed in our patient cohort without tumor tissue NGS analysis. We also note that it is difficult to attribute the low *KRAS* detection rate in our study to technological versus biological limitations. However, the yield of cell-free DNA may be useful in this regard: in the ctcDNA-based NGS assay, one of the four patients with tumor tissue *KRAS* mutations not present in the plasma had a low yield of cfDNA, suggestive of technological limitations. The other three patients with high cfDNA yields in plasma may therefore be patients for whom tumor cells were not actively shedding DNA. A larger sample size will be required to determine if there is a significant association between plasma cfDNA yield and the sensitivity of detection of tumor mutations. In addition to the low sensitivity for tumor mutations that should be detectable by the blood-based NGS assay, there is a further limitation in that deletions, indels, and rearrangements cannot be detected by the blood-based NGS assay (but can on the tumor tissue NGS assay).

A possible explanation for the low sensitivity is the heavily pre-treated nature of our patient population: nearly all of our patients had received some form of systemic chemotherapy prior to blood-based NGS analysis. Chemotherapy likely has a strong impact on the levels of cfDNA, making the detection of mutated cfDNA difficult in the setting of active therapy, irrespective of the current extent of radiographic response at the time the blood sample was obtained. Follow-up studies could include a stratified population of stage-matched patients comprised of treatment-naïve patients, patients responding to therapy and patients progressing on first line therapy or beyond.

Our observation that the presence of tumor DNA in cfDNA is a negative prognostic factor is consistent with numerous other studies. Multiple studies using digital PCR have demonstrated that high levels of circulating *KRAS* mutations in pancreatic cancer patients adversely affect survival [24], [25]. Another group used digital PCR to detect alterations in *KRAS*, *BRAF*, and *PIK3CA* in pancreatic cancer patients, demonstrating that detection of any of these genes was associated with lower progression-free survival [26]. The level of mutated cfDNA is prognostic in other cancer types as well, with higher levels of mutated *KRAS* and *BRAF* DNA found in colorectal cancer patients with lower survival [27].

The low concordance between tumor tissue and cfDNA-based NGS that we observed here indicates that further technical development is needed in this specific setting of late-stage pancreatic cancer. Nonetheless, despite the limitations we observed, we have demonstrated that it is feasible to utilize blood-based NGS profiling across many different hospitals and community-based practices; this is in contrast to other similar studies that were conducted at large academic institutions [11, 26]. The ability to use the extremely high KRAS mutation frequency in pancreatic cancer provided us with an important control to directly assess technical feasibility of cfDNA based molecular profiling of metastatic cancer patients where access to tumor tissue may problematic. Moreover, the ability to measure genomic alterations in patient matched cfDNA and tumor tissue in consecutively consented patients that are enrolling in an “all comers” national program that closely mimic what would be expected be seen at any point in time in clinics and physician offices around the United States provides a unique assessment window. Increasing analytical sensitivity using techniques such as dPCR could certainly increase concordance rates and identify molecular alterations where cfDNA concentrations are low in any given patient. However at this time, technologies like dPCR are not readily available in the community setting, where most patients are being treated. In the future, an optimized version of these platforms may allow for application of a personalized therapeutic approach to a greater number of pancreatic cancer cases, particularly those in which a tumor biopsy is hard to obtain.

## MATERIALS AND METHODS

### Patients

Pancreatic cancer patients were recruited under an IRB-approved registry into a molecular profiling program after obtaining informed consent. Patients from 18 different hospitals/high volume cancer centers and 7 different community oncology practices were enrolled and data collected in the registry. Patients were enrolled sequentially, during the time frame of this Pilot program.

### Next-generation sequencing of circulating DNA

Whole blood samples were sent to one of two commercial laboratories for targeted NGS analysis. The Guardant360 test (Guardant Health, Redwood City, CA) involves targeted sequencing of a 68-gene panel (Table S3B) in cfDNA using the Illumina HiSeq 2500 platform as previously described [28], whereas the ClearID test (Cynvenio, Westlake Village, CA) involves targeted sequencing of a 50-gene panel (Table S3C) in ctcDNA using the IonTorrent PGM platform. In the Guardant360 test, 20 mL whole blood is stabilized in cell-free DNA BCT tubes (Streck, Omaha, NE), which prevent lysis of blood cells for up to seven days [29]. In the ClearID test, 20 mL whole blood is collected into K_2_EDTA tubes and then stabilized in a proprietary fixative, which allows for a window of four days between sample collection and analysis. The Guardant360 assay generates read depths above 8, 000x and has a detection limit for frequencies of 0.25% [10]. At an optimal DNA input of 10 ng and minimum read depth of 500x, the ClearID test is validated for detecting variants at allele frequencies as low as 1% [30]. In all but five cases, the labs received the samples one day after collection; four samples were in transit for two days, and one sample was in transit for four days.

### Next-generation sequencing of tumor tissue

Formalin-fixed, paraffin-embedded tumor tissue samples were sent to a commercial laboratory for NGS testing (FoundationOne, Foundation Medicine, Cambridge, MA), which targets a 321-gene panel (Table S3A) for sequencing on the Illumina HiSeq 2500 platform. The overlap between panels was high: 65/68 (95.6%) genes from the Guardant360 panel were present on the FoundationOne panel, while 49/50 (98%) genes from the ClearID panel were present on the FoundationOne panel.

### Statistical analysis

All statistical analysis was performed in R. Survival differences between patient groups were determined using the log-rank test on Kaplan-Meier curves with the R *survival* package [31]. When performing survival analysis, follow-up time was defined as the number of weeks since blood sampling.

### Assay concordance

The concordance between NGS assays was evaluated by calculating the gene-level sensitivity and precision of blood-based NGS assays in detecting mutations present in the tumor tissue, as identified by NGS. A gene variant was considered concordant if the exact nucleotide change was present in the same gene and patient in both blood-based and tumor tissue biopsy.

## SUPPLEMENTARY MATERIALS FIGURES AND TABLES




