# Supplementary material for: A pilot study evaluating concordance between blood-based and patient-matched tumor molecular testing within pancreatic cancer patients participating in the Know Your Tumor (KYT) initiative
Source: Oncotarget. 2016 Nov 8;8(48):83446–56. doi: 10.18632/oncotarget.13225 (PMC5663527; doi:10.18632/oncotarget.13225)
Supplement: Supplementary file 1 [file oncotarget-08-83446-s001.pdf]

A pilot study evaluating concordance between blood-based and patient-matched tumor molecular testing within pancreatic cancer patients participating in the know your tumor (KYT) initiative

Supplementary Material

Supplemental Figure S1

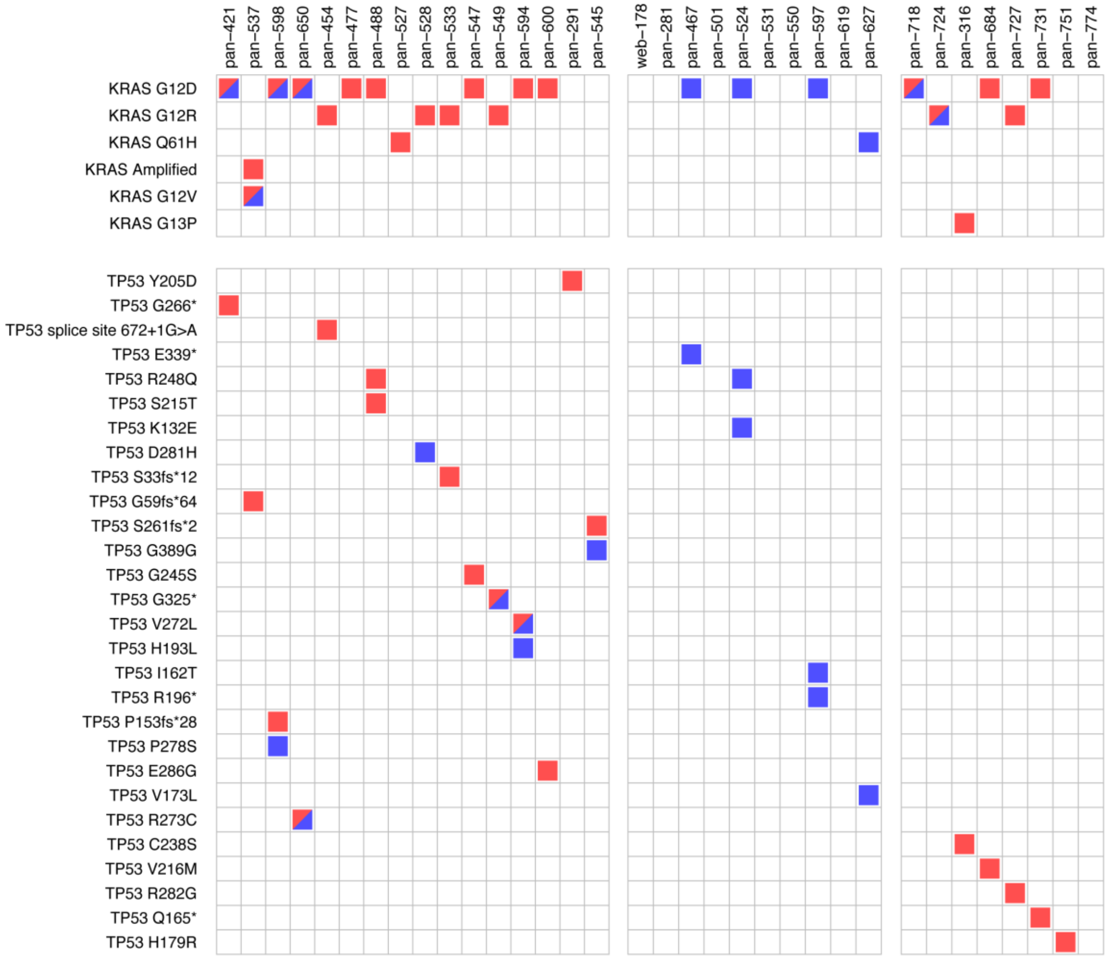

Supplemental Figure S1: Amino acid-level changes in *KRAS* and *TP53*. The specific *KRAS* (top block) and *TP53* (bottom block) amino acid substitutions are listed in the rows and the patient IDs are listed in the columns. Patients are ordered as in Figure 3 from the main text, with the left block comprising patients with both tumor and cfDNA NGS results, the middle block comprising patients with cfDNA NGS results only, and the right block comprising patients with both tumor and ctcDNA NGS results. Red cells indicate a mutation was detected in the tumor NGS assay only, blue cells indicate a mutation was detected in the cfDNA/ctcDNA NGS assay only, and the red/blue cells indicate that a mutation was concordant in both tumor and cfDNA/ctcDNA NGS assays.

Supplemental Figure S2

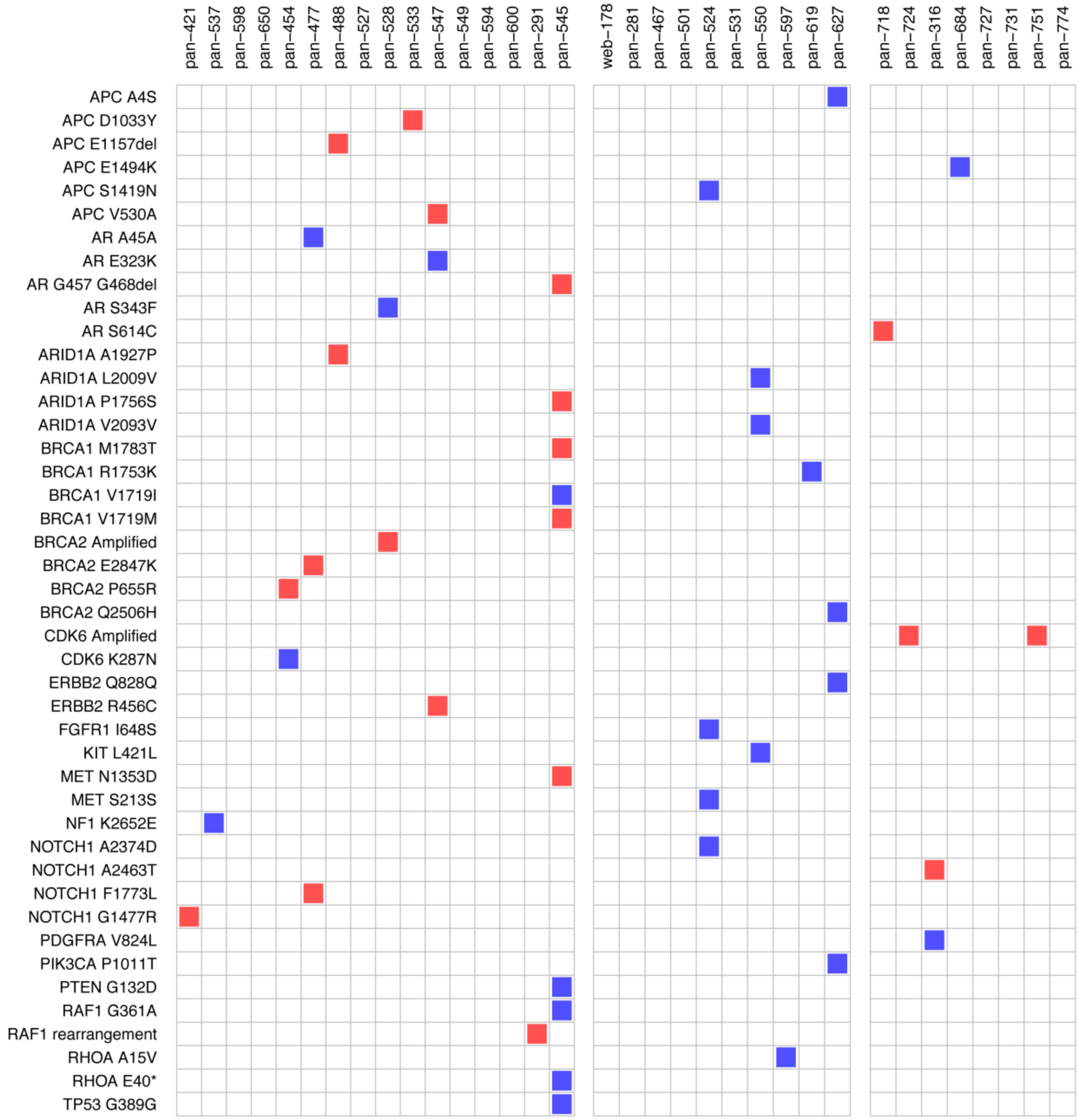

Supplemental Figure S2: VUS differences between liquid and tumor biopsies. Potentially actionable amino acid substitutions are listed in the rows and the patient IDs are listed in the columns. Patients are ordered as in Figure 3 from the main text, with left block comprising patients with both tumor and cfDNA NGS results, the middle block comprising patients with cfDNA NGS results only, and the right block comprising patients with both tumor and ctcDNA NGS results. Red cells indicate a mutation was detected in the tumor NGS assay only and blue cells indicate a mutation was detected in the cfDNA/ctcDNA NGS assay only.

Supplemental Figure S3

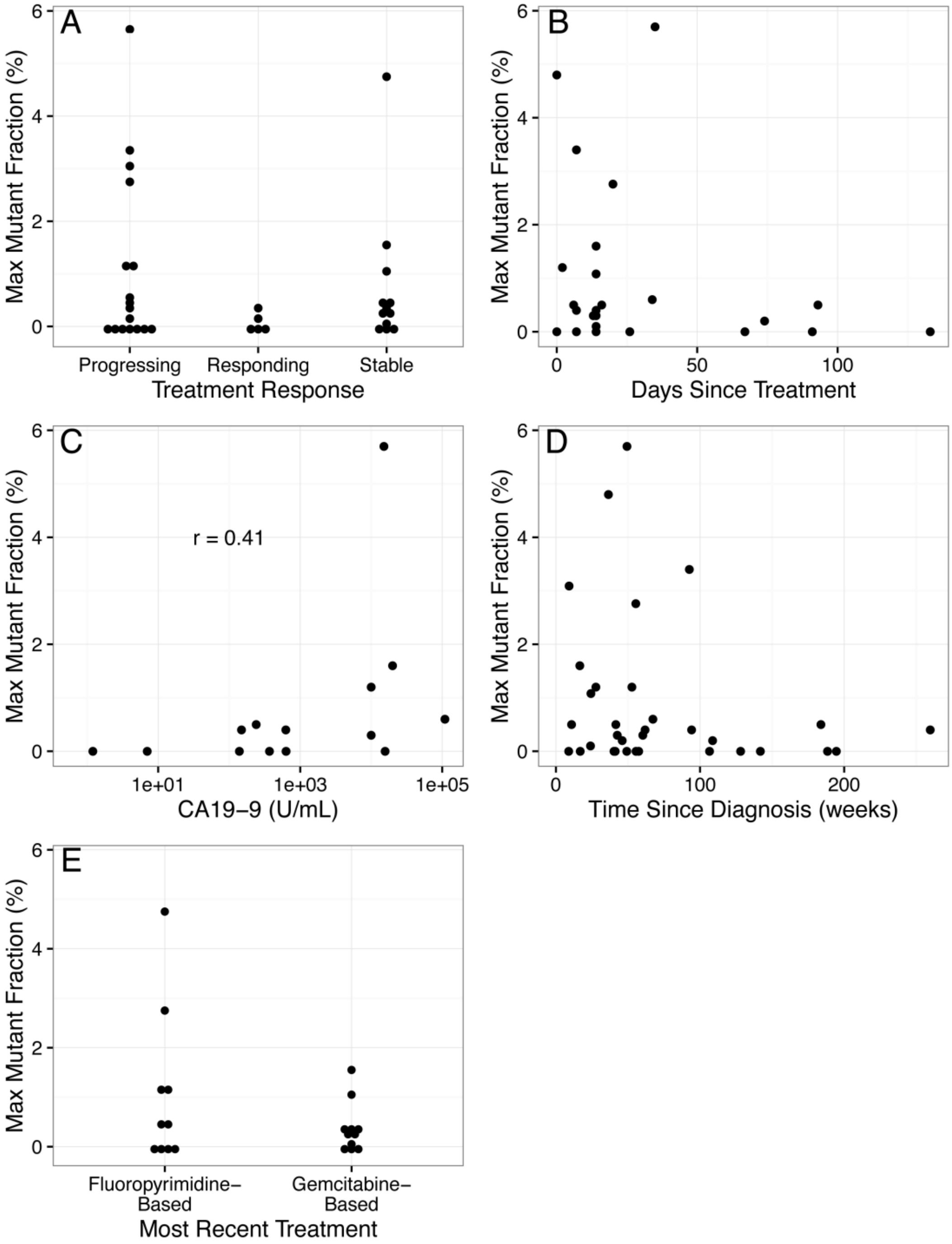

Supplemental Figure S3: Relationships between maximum mutant allele fraction in cfDNA and clinical variables. Overall somatic burden in the blood, assessed by the maximum mutant allele fraction, was compared to current treatment response status **(A)**, whether a treatment was administered in the month prior to liquid biopsy **(B)**, the most recent CA19-9 measurement **(C)**, the time since initial pancreatic cancer diagnosis **(D)**, and the most recent treatment type **(E)**. The most recent CA19-9 measurements were

only plotted in patients for whom the CA19-9 blood draw occurred less than one month before or after the liquid biopsy blood draw (N = 12). The most treatment types were only plotted in patients for whom the treatment was administered less than one month prior to the liquid biopsy blood draw (N = 17). Fluoropyrimidine-based treatments were those containing either 5-fluorouracil or capecitabine.
